# Transcription Factor Engineering for High-Throughput Strain Evolution and Organic Acid Bioproduction: A Review

**DOI:** 10.3389/fbioe.2020.00098

**Published:** 2020-02-19

**Authors:** Jia-Wei Li, Xiao-Yan Zhang, Hui Wu, Yun-Peng Bai

**Affiliations:** State Key Laboratory of Bioreactor Engineering, East China University of Science and Technology, Shanghai, China

**Keywords:** transcription factor, biosensor, metabolic engineering, synthetic biology, organic acid, high-throughput screening

## Abstract

Metabolic regulation of gene expression for the microbial production of fine chemicals, such as organic acids, is an important research topic in post-genomic metabolic engineering. In particular, the ability of transcription factors (TFs) to respond precisely in time and space to various small molecules, signals and stimuli from the internal and external environment is essential for metabolic pathway engineering and strain development. As a key component, TFs are used to construct many biosensors *in vivo* using synthetic biology methods, which can be used to monitor the concentration of intracellular metabolites in organic acid production that would otherwise remain “invisible” within the intracellular environment. TF-based biosensors also provide a high-throughput screening method for rapid strain evolution. Furthermore, TFs are important global regulators that control the expression levels of key enzymes in organic acid biosynthesis pathways, therefore determining the outcome of metabolic networks. Here we review recent advances in TF identification, engineering, and applications for metabolic engineering, with an emphasis on metabolite monitoring and high-throughput strain evolution for the organic acid bioproduction.

## Introduction

In nature, transcription factors (TFs) control the rate of gene transcription by recognizing specific DNA sequences, thus regulating expression of the genome. In addition to the normal biological and physiological roles that TFs play in human cells, they can be used as building blocks and regulatory tools in metabolic engineering and synthetic biology (Yadav et al., 2012; Shi et al., 2018; Yang et al., 2019). For example, in one study 55 TFs and 750 metabolic genes were used to construct a regulatory network for controlling metabolism in *Saccharomyces cerevisiae* (*S. cerevisiae*) (Herrgård et al., 2006). There are a wide range of TFs available from diverse microbes, and TF engineering is a very flexible approach, which makes TFs a particularly useful resource for biotechnology. Functional screening is used to identify novel TFs that can then be engineered in host cells to control the expression of key enzymes in biosynthetic gene clusters (BGCs) (Liu et al., 2013). In past decades, researchers have designed and optimized many biosynthetic pathways for natural products (Yadav et al., 2012), such as artemisinin (Paddon and Keasling, 2014). When constructing such pathways, over-expressing positive regulators or knocking down/out negative regulators are two important ways of activating BGCs (Xie et al., 2015; Thanapipatsiri et al., 2016). As an example, over-expressing the global TF AdpA in *Streptomyces hygroscopicus* enhances gene cluster transcription and antibiotic synthesis (Tan et al., 2013). Novel genomic editing and protein engineering tools have been applied to synthesize target products via TF-mediated activation of silent BGCs (Tong et al., 2015; Zhang et al., 2017; Grau et al., 2018). Because TFs are sensitive to their corresponding signal molecules, they can also be used to construct highly sensitive biosensors for use in high-throughput screening for improved strains (Yu et al., 2019). Protein engineering can be used to alter the ligand specificity of TFs such that they can detect new signaling molecules (Machado et al., 2019), furthering expanding their applications in metabolic engineering.

With the rapid development of bioinformatics and genomic editing tools, TFs are playing more important roles in improving the microbial production of valuable chemicals. In this paper, we will briefly review recent advances in the use of microbial TFs to regulate metabolic production of valuable chemical products, with a particular focus on the production of organic acids. We will also summarize strategies for identifying new TFs and review the use of TFs as biosensors for monitoring metabolites *in vivo* and performing high-throughput screening for overproducers, which are important methods used to obtain a strain with the desired phenotype from a library of mutants containing a wide variety of genomic alterations.

## Methods for Identifying TFs

**FIGURE 1:**
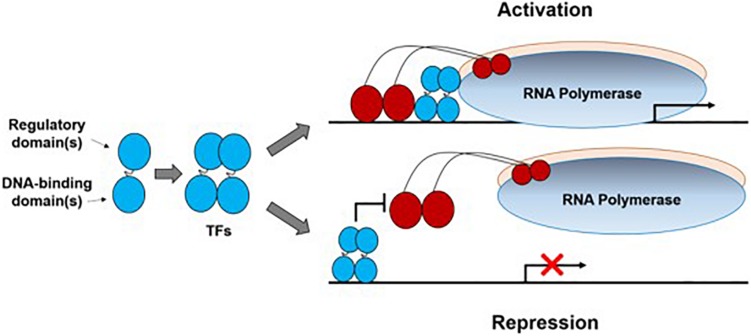
Illustration of bacterial transcription factors (TFs). A transcription factor subunit (denoted as a dumbbell shape) usually contains a regulatory domain and a DNA-binding domain. Normally two subunits dimerize to form a TF which interacts with a bacterial promoter region to either activate or repress transcription initiation. Here, only one activation mode (TF contacts domain 4 of the RNA polymerase σ subunit) and one repression mode (via steric hindrance) were shown to illustrate this process.

Transcription factors are sequence-specific DNA-binding proteins that bind to promoters to either activate or repress transcription (Figure 1). So far, TFs in prokaryotes can be grouped into a dozen families identified on the basis of sequence analysis, with the LacI, AraC, LysR, CRP, TetR, and OmpR families being characterized best (Browning and Busby, 2004). New TFs continue to be identified by experimental methods such as transcriptome analysis (Raghavan et al., 2019), one-hybrid assays (Reece-Hoyes and Walhout, 2012), electrophoretic mobility shift assay (EMSA, Hellman and Fried, 2007), DNA affinity purification-mass spectrometry (AP-MS, Tacheny et al., 2013), and protein microarrays (Hu et al., 2009).

In general, TFs have a DNA-binding domain (DBD) and a regulatory domain (RD). Knowledge of binding sites, ligand-protein interaction and binding affinity can help identify an unknown DNA-binding protein. DBDs have been widely characterized both experimentally and bioinformatically, so today, most newly discovered and putative TFs can be identified and grouped by sequence homology to a previously characterized DBD. Now, information on the DBD structures in complex with DNA can be found in the Protein Data Bank^1^. Bacterial TF binding sites and related information are also available in some open databases such as CollecTF (Kilic et al., 2014). RD, also called “effector binding domain,” performs many tasks including ligand binding, protein-protein interactions and modulating the DNA-binding affinity of TFs. The diversity, abundance and structure variability of RD have been identified and investigated systematically for transcription regulation (Perez-Rueda et al., 2018; Sanchez et al., 2020). In particular, the high variability of RDs and recognition promiscuity may have evolutionary implications that they can be targeted for engineering to change the ligand specificity and/or improve the sensing dynamics.

## Using TFs as Biosensors in Metabolic Engineering

### Monitoring Metabolites *in vivo*

**FIGURE 2:**
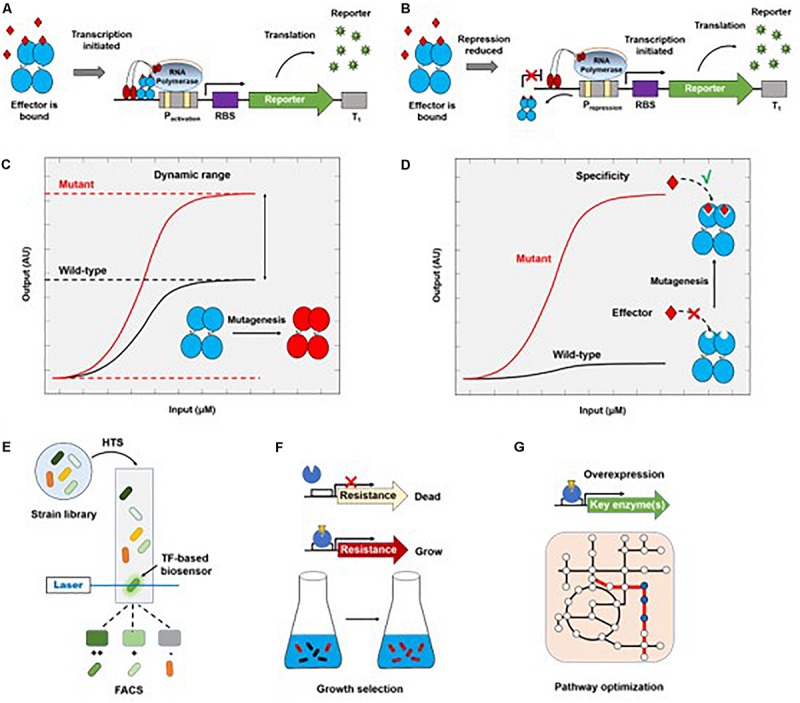
Genetically encoded TF-based biosensors and their applications in metabolic engineering. Metabolic molecules can be transformed into a detectable reporter molecule through the initiation of reporter transcription by an activator **(A)** or a repressor **(B)**. The correlation between input (product concentration) and output (AU, arbitrary reporter units) provides the dynamic range **(C)** and ligand specificity of transfer function **(D)**. The wild-type TFs can be engineered so that the mutant can have a higher dynamic range **(C)** or sense a new type of molecule **(D)**. TF-based biosensors can be coupled to HTS methods such as FACS **(E)** or to growth **(F)** for screening overproducers. TFs can also be engineered to optimize biosynthetic pathways of target products **(G)**.

Due to the inherent complexity of biological systems, it is desirable to quantitatively or semi-quantitatively evaluate metabolites of interest *in vivo*. By binding metabolites of interest, TFs can either activate the expression of reporter genes in a genetic circuit as an activator (Figure 2A), or induce the downstream gene expression by reducing the repression of RNA polymerase-promoter binding (Figure 2B), resulting in a detectable signal that can be easily assayed (Dietrich et al., 2010; Mahr and Frunzke, 2016; D’Ambrosio and Jensen, 2017; Liu Y. et al., 2017). TFs can be used to detect small molecules, ion accumulation, and changes in physiological parameters. A wide variety of TF-based biosensors have been constructed and characterized that recognize different small molecules, including but not limited to glutarate (Thompson et al., 2019), 3-hydroxypropionic acid (Rogers and Church, 2016; Seok et al., 2018; Nguyen et al., 2019), itaconic acid (Hanko et al., 2018), flavonoids (Siedler et al., 2014a; Trabelsi et al., 2018), anhydrotetracycline (Lutz and Bujard, 1997), arabinose (Lee and Keasling, 2006), lactam (Zhang et al., 2016), mevalonate (Tang and Cirino, 2011), L-methionine (Mustafi et al., 2012), amino acids (Binder et al., 2012; Mustafi et al., 2012; Leavitt et al., 2016), acrylic acid (Raghavan et al., 2019), isoprene (Kim et al., 2018), shikimate (Liu C. et al., 2017), and aromatic compounds (Willardson et al., 1998; Kim et al., 2005; Jha et al., 2016). Some typical biosensors with sensing kinetics are listed in Table 1.

**TABLE 1:** Overview of some TF-based biosensors with sensing kinetics.

**Host chassis**	**TFs**	**Analyte**	**Detection range**	**Reporter**	**References**
*E. coli*	LuxR	Butanoyl-homoserine lactone	10 nM-1 μm	GFP	Hawkins et al., 2007
E. coli XL1-Blue	BmoR	Isobutanol	0–100 mM	GFP	Yu et al., 2019
*Saccharomyces cerevisiae*	FapR	Glucose	95-fold to 2.4-fold	GFP	Dabirian et al., 2019a
*E. coli*	AraC	D-Arabinose	100 mM	GFP	Tang et al., 2008
*E. coli*	PobR	p-Nitrophenol	<2 μM	GFP	Jha et al., 2016
*Pseudomonas putida*	GcdR	Glutarate	2.5 mM	RFP	Thompson et al., 2019
*E. coli*	HucR	Shikimic Acid	3–20 mM	RFP	Li et al., 2017
*E. coli*	LysR	3-Hydroxypropionic acid	0.01–100 mM	GFP	Seok et al., 2018
*E. coli*	YpItcR	Itaconic acid	0.07–0.7 nM	RFP	Hanko et al., 2018
*E. coli DH5*	FdeR	Naringenin	100 μM	RFP	Trabelsi et al., 2018
*E. coli DH10B*	ChnR	Lactams	3–12.5 mM	RFP	Zhang et al., 2016
*E. coli*	AraC	Mevalonate	<1 mM	GFP	Tang and Cirino, 2011
*E. coli*	Lrp	L-Methionine	>0.2–23.5 mM	eYFP	Mustafi et al., 2012
*E. coli*	LysR	L-Lysine	<5 mM	eYFP	Binder et al., 2012
*E. coli*	PyHCN	Acrylic acid	500 μM	eGFP	Raghavan et al., 2019
*E. coli*	TbuT	Isoprene	0.1 mM	eGFP	Kim et al., 2018
*Corynebacterium glutamicum*	LysR	Shikimic acid	19.5–120.9 mM	eGFP	Liu C. et al., 2017

Although wild-type TFs are sensitive to their corresponding metabolites, they have narrow dynamic ranges, which limits their practical applications. Thus, TFs are often engineered to increase their dynamic ranges (Figure 2C). LuxR, a TF that is involved in quorum sensing in many bacteria, has been engineered to respond to butanoyl-homoserine lactone at concentrations as low as 10 nM (Hawkins et al., 2007). Structural analysis and site-directed mutagenesis were used to engineer a BmoR mutant that has a wider detection range (0–100 mM) for intracellular isobutanol than the wild-type protein (Yu et al., 2019). Promoter binding sites can also influence the dynamic range of TFs. The maximum dynamic range of a bacterial TF-based biosensor in *S. cerevisiae* was expanded by modifying promoter sequences (Dabirian et al., 2019a). Leavitt et al. (2016) engineered both wild-type TFs and promoters involved in aromatic amino acid induction and regulation in S. cerevisiae to obtain a transcriptional output 15-fold greater than the off-state. Similarly, TyrR, a TF that is activated in response to increased intracellular L-Phe concentrations in *E. coli*, exhibited higher sensitivity when combined with optimized promoters, with a dynamic range up to 15 times greater than when it was used in a strain with wild-type promoter sequences (Liu Y.F. et al., 2017).

In addition to naturally occurring metabolite-responsive biosensors, there are many metabolites for which a natural TF does not exist, or for which the detection limit is too high. To address this problem, a known TF, such as PcaV (Machado et al., 2019), can be engineered by directed evolution to expand is sensing profile (Figure 2D). Another example is the switch in the effector specificity of an *L*-arabinose-responsive TF, AraC, which was modified by molecular evolution to respond to D-arabinose concentrations as low as 1 mM (Tang et al., 2008). Biosensor engineering by random domain insertion (BERDI) is another technique that can be used to generate new metabolite-responsive TFs. In this case, *in vitro* transposon-mediated mutagenesis was used to construct a TF library, followed by fluorescence-activated cell sorting (FACS) to isolate functional biosensors (Younger et al., 2017). Recently, MphR was found to bind promiscuously to macrolides, and was then engineered to improve its sensitivity, specificity, and selectivity for these small molecules (Kasey et al., 2017). The tailored MphR biosensors provide a useful means of screening key enzymes involved in complex macrolide biosynthesis, demonstrating the power of protein engineering in creating new metabolite-responsive TFs. Similarly, an *Acinetobacter* TF, PobR, was engineered to switch its specificity from the native effector 4-hydroxybenzoate to p-nitrophenol (pNP) (Jha et al., 2016). Given the significant similarity between the two effectors, the high specificity of this engineered TF for pNP (detection limit of 2 μM) demonstrates the importance of engineering TFs by directed evolution.

### TF-Biosensor Based Strain Screening

Since only a few metabolites are natural chromophores or fluorophores, high-throughput screening (HTS) methods are needed to identify engineered microorganisms that produce the desired products. The application of TF-based biosensors to high-throughput screening has recently been reviewed in detail (Bott, 2015; Mahr and Frunzke, 2016; Cheng et al., 2018). Here, we focus on combining random genomic mutation with high-throughput screening to obtain high-yield strains. Cells contain sophisticated metabolic networks, which can make it challenging to rationally engineer metabolic pathways that produce large amounts of target compounds. Although genomic editing tools enable the rapid and precise tuning of gene expression, our ability to rewire cellular metabolism is still limited (Yadav et al., 2012). Therefore, genome-wide approaches for introducing random mutations, followed by high-throughput screening, provide an efficient way to evolve strains (Figure 2E). Chou and Keasling (2013) developed a feedback-controlled system that contains different TFs for sensing isopentenyl diphosphate in bacteria and yeast. Several rounds of adaptive laboratory evolution (ALE) resulted in a strain that produces more tyrosine and isoprenoid than the original strain (Chou and Keasling, 2013). Recently, a cooperative two-factor ALE was developed to enhance lipid production in *Schizochytrium sp.* by 57.5% relative to the parent strain after 30 adaption cycles (Sun et al., 2018).

Transcription factor-based biosensors turn a chemical input, which normally does not have an observable phenotype, into a detectable output, such as fluorescence, which can be easily coupled to FACS, an ultra-throughput method capable of screening of more than 50,000 cells per second, thereby greatly accelerating the optimization of production strains. The power of TF sensor–based FACS screening is clear. For example, using an L-lysine sensor, a library of 10 million mutated *E. coli* cells was screened by FACS in 30 min (Wang Y. et al., 2016). The best mutant was selected and evaluated in a 5-L fermenter within 2 weeks after one round of HTS, which is 10^4^–10^5^ times faster than traditional selection methods. The number of studies that have applied this strategy is increasing rapidly (Binder et al., 2012, 2013; Mustafi et al., 2012; Siedler et al., 2014b, 2017; Jha et al., 2016; Wang Y. et al., 2016; Liu Y.F. et al., 2017; Schulte et al., 2017; Flachbart et al., 2019; Kortmann et al., 2019). In practice, cross-talk between cells should be noted, as it may lead to false positive results, decreasing the screening efficiency; this can be minimized by optimizing expression and cultivation conditions (Flachbart et al., 2019).

In contrast to the use of fluorescence-coupled biosensors, growth-coupled screening is a high-throughput method that can be performed without expensive equipment (Figure 2F; Dietrich et al., 2010). Lee and Oh (2015) developed a suicide riboswitch, *glmS*, for the high-throughput screening of metabolites in *S. cerevisiae*. Growth of the strain harboring the suicide riboswitch was restored when the level of the metabolite of interest level increased. An N-acetyl glucosamine producer strain was isolated after screening. Liu S.D. et al. (2017) coupled the microbial production of L-tryptophan (L-Trp) to cell growth with maltose as the sole carbon source. The selection of mutated producers led to a strain with up to 65% increased L-Trp production. An approach combining growth recovery with a fluorescent reporter protein has also been developed for enzyme-directed evolution (Michener and Smolke, 2012).

## TF Engineering for the Microbial Production of Organic Acids

Organic acids and their derivatives have a wide range of industrial applications, and can be used as food additives, pharmaceuticals, antimicrobial agents, biomaterials, biofuels, and more (reviewed in Chen and Nielsen, 2016; Liu J.J. et al., 2017). Due to recent concerns about climate change and limited fossil reserves, the use of renewable biomass for the biological production of fuels and chemicals has received increasing attention as an alternative to chemical production (Sarria et al., 2017). Large-scale production using microorganisms requires the development of HTS tools for strain engineering and techniques for analyzing strain performance and the efficiency of biological processes. Many TF-based biosensors for measuring the intracellular concentrations of organic acids are currently available (Sint Fiet et al., 2006; Uchiyama and Miyazaki, 2010; Dietrich et al., 2013; Tang et al., 2013; Raman et al., 2014; Chen et al., 2015; Zhou et al., 2015; Leavitt et al., 2017; Liu C. et al., 2017; Hanko et al., 2018; Nguyen et al., 2019; Raghavan et al., 2019; Thompson et al., 2019) that provide a HTS method when combined with FACS. Alternatively, TFs can activate or repress the expression of target genes, which can be used to rewire microbial metabolic pathways, thus leading to an increase in the production of organic acids (Figure 2G). Compared with HTS-based strain evolution, engineering TFs for pathway reconstruction requires extensive knowledge of cellular metabolic machinery. Here, we discuss some recent examples of studies that have improved organic acid production using the above two strategies, with an emphasis on the role TFs play in this process (Table 2).

**TABLE 2:** Strain evolution for the enhanced production of organic acids.

**Host chassis**	**TF**	**Screening/Analysis**	**Method**	**Product**	**Enhancement**	**References**
*E. coli*	LysR	Growth selection	Enzyme directed evolution	3-HP	2.79-fold in catalytic efficiency of α-ketoglutaric semialdehyde dehydrogenase	Seok et al., 2018
*E. coli*	SoxR	FACS	Genome-wide mutagenesis by CRISPR	3-HP	7- and 8-fold increase in productivity	Liu et al., 2018
*E. coli*	PyHCN	FACS	Enzyme directed evolution	Acrylic acid	50% increase in catalytic efficiency of an amidase	Raghavan et al., 2019
*E. coli JM109*	HIF-1	HPLC	TF engineering	Pyruvic acid	Titer increased to 53.1 g/L	Luo et al., 2020
*E. coli*	LysR	HPLC	TF engineering	Itaconic acid	215-fold in itaconic acid detection	Hanko et al., 2018
*E. coli*	ARO1	FACS	Combined ALE and metabolic engineering	Muconic acid	Titer increased to 2.1 g/L	Leavitt et al., 2017
*Corynebacterium glutamicum*	ShiR	FACS	RBS engineering	Shikimic acid	Titer increased to 3.72 mM	Liu C. et al., 2017
*E. coli*	pfkA	HPLC	Dynamic control of metabolic fluxes	D-Glucaric acid	Titer improved by up to 42%	Reizman et al., 2015
*E. coli*	acuR/prpR	FACS	Design–build–test cycle	3-HP	Titer increased to 4.2 g/L	Rogers and Church, 2016
*S. cerevisiae*	FadR	FACS	Gene library	Fatty acid	30% increased fatty acid level observed	Dabirian et al., 2019b

### 3-Hydroxypropionic Acid (3-HP)

3-Hydroxypropionic acid is a platform molecule for the production of 3-carbon chemicals; in particular, it can easily be converted to acrylic acid upon dehydration. However, it is difficult to detect intracellular 3-HP. To address this problem, Rogers and co-workers developed a system that uses the TF CdaR to generate a fluorescent readout in proportion to the intracellular concentration of a target metabolite (Rogers and Church, 2016). Using this sensor, the authors were able to identify a strain that produced 4.2 g/L 3-HP, a level that is 23-fold higher than any previously reported. Liu et al. (2018) introduced genome-wide mutations to target 30 genes including a TF SoxR that plays important roles in genome-level transcription. The mutant SoxR_S__26__G,E__32__V_ conferred high furfural and acetate resistance to the engineered strain, leading to a 7- and 8-fold increase in 3-HP productivity relative to the parent strain under high furfural and high acetate hydrolyzate fermentation, respectively, demonstrating the importance of the TF-mediated global regulation of gene expression.

### Acrylic Acid (AA)

As mentioned above, AA biosynthesis via enzymatic dehydration of 3-HP has been demonstrated in engineered *E. coli* (Chu et al., 2015; Liu and Liu, 2016). However, the yields are low, which led Raghavan et al. (2019) to apply HTS to identify strains the exhibited greater activity of key enzymes in the AA synthesis pathway. By identifying *E. coli* genes that were selectively up-regulated in the presence of AA, this group found that the gene *yhcN* encodes a protein that can respond specifically to AA at low concentrations when it was coupled to an eGFP reporter (Raghavan et al., 2019). This biosensor was used to find an amidase variant that converted acrylamide to AA with 1.6-fold improvement in catalytic efficiency, which is important to enhance AA production.

### Pyruvic Acid (PA)

Pyruvic acid is widely used as additive in food and cosmetics, and as a starting material for the biosynthesis of pharmaceuticals such as L-tyrosine and (*R*)-phenylacetylcarbinol (Song et al., 2016). Currently, the microbial production of PA from renewable biomass requires high levels of dissolved oxygen (DO). To remove this restriction, hypoxia-inducible factor 1 (H1F-1) was engineered to increase the transcription of key enzymes involved in PA synthesis under low DO levels (Luo et al., 2020). The stability of H1F1 was further optimized, resulting in a titer of 53.1 g/L for PA production in a 5-L bioreactor under 10% DO.

### Itaconic Acid (IA)

As an unsaturated 5-carbon dicarboxylic acid, IA is a useful monomer for constructing synthetic polymers. IA can also be used as a precursor for the production of high-value chemicals (Thakker et al., 2015). Variants of a number of microorganisms, such as *Aspergillus terreus*, *E. coli*, and *S. cerevisiae*, have been developed that produce IA. To further improve production titers, it is important to be able to quantify intracellular levels of IA. Recently, Hanko et al. (2018) reported the development of the first IA biosensor based on identifying LysR-type TFs and their promoters in *Yersinia pseudotuberculosis* and *Pseudomonas aeruginosa*. The *Yp*ItcR/P_*ccl*_ inducible system was used in *E. coli* to identify the optimum expression level of a key enzyme in the IA synthesis pathway. The dynamic range was 5–100 μM, which could be improved further by protein engineering. This biosensor displayed the potential to facilitate improved IA biosynthesis through high-throughput strain development.

### Muconic Acid (MA)

Microbial production of MA was first demonstrated in *E. coli* (Niu et al., 2002), and achieved a level of 18 g/L. Later, to simplify downstream separations and reduce high alkali loads, *S. cerevisiae* was used to produce MA at a low pH, although the titers were low (Weber et al., 2012; Curran et al., 2013). By combining metabolic engineering and electrocatalysis, Suastegui et al. (2016) were able to engineer a strain that produces an MA titer of nearly 560 mg/L. To further improve the productivity, Leavitt et al. (2016, 2017) applied a combined ALE and rational engineering strategy, in which an aromatic amino acid (AAA) biosensor was coupled to anti-metabolite selection to increase the activity of the AAA biosynthetic pathway. Activating this pathway led to a significant improvement in MA production titer to 2.1 g/L in a fed-batch bioreactor, representing the highest titer obtained to date.

### Shikimic Acid (SA)

Shikimic acid is an important metabolic intermediate of the shikimate pathway. Various microorganisms have been engineered to produce SA (Licona-Cassani et al., 2014; Martínez et al., 2015; Kogure et al., 2016). Liu C. et al. (2017) developed an SA biosensor constructed from a LysR-type transcriptional regulator ShiR to monitor the SA production of different *Corynebacterium glutamicum* strains (Schulte et al., 2017). This biosensor was used to identify a high-yield SA-producing strain with 2.4-fold improvement in titer over low-yield strains identified by FACS. Taking another approach, Li et al. (2017) performed directed evolution of a uric acid–responsive regulatory protein, HucR, to switch its specificity to SA, which the mutant sensor can detect in the range of 3–20 mM. The biosensor was used to monitor metabolic flux and improve the specific activity of a key enzyme in the SA biosynthetic pathway.

### Glucaric Acid (GA)

Glucaric acid is a promising platform molecule for making synthetic polymers such as nylons and plastics. The microbial production of GA was first demonstrated in *E. coli*, and subsequently developed strains produce titers of up to 4.85 g/L (B strain) and 2 g/L (K strain) (Moon et al., 2009, 2010; Shiue and Prather, 2014; Reizman et al., 2015; Doong et al., 2018; Qu et al., 2018). More recently, to overcome the limitation of acid-induced toxicity, an *S. cerevisiae* strain was engineered to produce GA (Gupta et al., 2016; Chen et al., 2018). Raman et al. (2014) described the development of a selection system that uses a biosensor to couple metabolite concentrations to cell fitness. A negative selection scheme was also developed to rule out false positives. After four rounds of evolution, GA production was increased 22-fold, although the absolute titer was lower than that produced by the *E. coli* K strain. Later, another group developed a general GA-responsive biosensor (Rogers and Church, 2016). Recently, Zheng et al. (2018) employed this biosensor to construct a two-strain system for rapid screening of myo-inositol oxygenase mutants, which play a key role in the GA synthesis pathway (Zheng et al., 2018), and found that fine-tuning the cofactor balance resulted in an increase in GA production in yeast.

### Fatty Acid (FA)

Due to the lack of techniques available for monitoring fatty acyl-CoA levels *in vivo*, historically it has been very challenging to design rational approaches to identifying genes that modulate the production of these compounds. Recently, a FadR-based biosensor was developed to screen for *S. cerevisiae* genes that increase the fatty acyl-CoA pool using FACS (Dabirian et al., 2019b). Using this biosensor, this group found that the overexpression of *GGA2* could increase fatty acid levels by 30 and 24% at 8 and 24 h after inoculation, respectively, which was mainly due to a significant increase in the C_16__:__1_ and C_16__:__0_ fatty acid levels. In addition, Bergman et al. (2019) used to HTS to find that overexpressing the TF Stb5 can enhance FA production in *S. cerevisiae*. This increase in FA production could be because of the consumption of excess NADPH, which would alleviate a potential redox imbalance.

## Conclusion and Perspective

In a context of growing concerns regarding climate change, environmental protection, and sustainable development, the biological production of chemicals, pharmaceutical products, fuels, and materials through microbial fermentation of renewable biomass has developed rapidly in the past decade, providing promising solutions for these issues. To make this approach economically feasible in practice, however, the titer, yield, and productivity of organic acid bioproduction, for example, need to be improved further (Chen and Nielsen, 2016). As a metabolite sensor and gene expression regulator, TFs play an important role in determining the end-product productivity in a cell factory. Therefore, the importance of TFs engineering is that it is a critical tool in optimizing phenotypes. In particular, the explosive development of genomic editing tools in a wide variety of prokaryotic and eukaryotic microorganisms since 2012 (reviewed in Csörgő et al., 2016; Wang H.F. et al., 2016; Tian et al., 2017; Shapiro et al., 2018), together with various high-throughput strategies for mutagenesis, screening, sorting, and sequencing, has facilitated and accelerated the strain improvement significantly. Researchers have begun to engineer TFs systematically to improve bioproduction efficiency. TFs are key components used to construct synthetic genetic circuits *in vivo*, and can be used to detect intracellular metabolites and even the activity of entire pathways that would otherwise remain “invisible.” TF-based biosensors also provide a HTS method for use in rapid strain evolution, as they enable overproducers to be identified quickly from a genome-wide mutant library using FACS and other technologies (e.g., microfluidics). The combination of genome-wide genomic editing and HTS technologies has drawn increasing attention in the field of strain development. In addition, TFs are also important global regulators that control the expression levels of key enzymes in biosynthesis pathways, therefore determining the direction, flux, balance, and outcome of metabolic networks. Identifying, engineering, and using TFs for applications such as biosensors can help fine-tune gene expression, improve the activity and stability of key enzymes, and direct metabolic flux, thus providing flexible tools for metabolic engineering, as has been demonstrated in the many works reviewed above.

Although there are many examples of successful TF engineering in metabolic engineering and synthetic biology, some challenges remain. To date, the majority of TF-based biosensors have been demonstrated in “proof-of-concept” experiments, with few examples showing real improvement in bioproduction. This is because the type, function, performance, specificity and number of TFs are still limited compared with the large number of host cells, pathways, and metabolites that need to be engineered. The development of novel, high-quality TFs with more functions relies on the further utilization of advanced bioinformatics, computational biology, and protein engineering. In addition, the molecular mechanism, compatibility, robustness, interaction, and quantification of heterogeneous TFs in regulating metabolic networks in host cells need to be understood and thoroughly elucidated to enhance the efficiency of TF-based strain development.

## Author Contributions

J-WL, X-YZ, HW, and Y-PB participated in searching and analyzing literature for this review, as well as writing and critical reviewing the manuscript.

## Conflict of Interest

The authors declare that the research was conducted in the absence of any commercial or financial relationships that could be construed as a potential conflict of interest.

## Footnotes

1 https://www.rcsb.org
